# Acute aortoiliac occlusive disease during percutaneous transluminal angioplasty in the setting of ST-elevation myocardial infarction: a case report

**DOI:** 10.1186/s13256-017-1544-4

**Published:** 2018-01-11

**Authors:** Anthony H. Kashou, Nabil Braiteh, Ali Zgheib, Hisham E. Kashou

**Affiliations:** 1 SUNY Upstate Medical University, 750 East Adams St, Syracuse, NY 13210 USA; 2 Department of Internal Medicine, Wilson Regional Medical Center, United Health Services Hospitals, 33-57 Harrison St, Johnson City, NY 13790 USA; 3 American University of Beirut, Maamari Street, Beirut, Lebanon; 4 Department of Cardiology, Wilson Regional Medical Center, United Health Services Hospitals, 30 Harrison St #250, Johnson City, NY 13790 USA

**Keywords:** Aortoiliac occlusive disease, Leriche syndrome, ST-elevation myocardial infarction, Percutaneous angioplasty, STEMI

## Abstract

**Background:**

Aortoiliac occlusive disease, which is also referred to as Leriche syndrome, is a chronic atherosclerotic occlusive disease that occurs at the level of the aortic bifurcation. It is often thought to present with a triad of clinical symptoms: (1) intermittent lower extremity vascular claudication, (2) impotence, and (3) weak/absent femoral pulses.

**Case presentation:**

We report a case of a 47-year-old Caucasian woman who presented with an acute inferior ST-elevation myocardial infarction. During percutaneous transluminal angioplasty, our patient suddenly developed severe bilateral lower extremity pain, absent femoral pulses, and cool extremities. Distal aortogram revealed 95% stenosis with an apple core-like lesion in the mid-abdominal aorta. Stent placement resulted in improved blood flow to the distal aortic segment and resolution of symptoms.

**Conclusions:**

The presence of significant peripheral vascular disease, significant cardiac risk factors, and/or difficulty accessing the femoral artery should caution a transfemoral approach during percutaneous transluminal angiography. This approach may precipitate aortoiliac occlusion and/or thromboembolism to the lower extremities. We encourage interventional cardiologists to (1) take extra caution when manipulating the wire and catheter and (2) strongly consider using a transradial approach in such patients.

## Background

Aortoiliac occlusive disease, which is also referred to as Leriche syndrome, results from a chronic obstructive process in the infrarenal aorta and iliac arteries [1–3]. It originates at the distal aorta or common iliac arteries from atheromatous plaque formation and progressively worsens over time to cause occlusion at the aortic bifurcation [1]. This chronic disease often takes decades to develop. It has a propensity to affect males with peripheral arterial disease in their third to sixth decade of life [2]. Patients often have associated cardiac risk factors such as hypertension, hyperlipidemia, diabetes mellitus, and tobacco abuse [4]. Leriche syndrome often presents with a triad of clinical symptoms: (1) intermittent lower extremity vascular claudication, (2) impotence, and (3) weak/absent femoral pulses [2, 5]. In this report, we discuss an atypical presentation of acute aortoiliac occlusive disease in a 47-year-old woman in the setting of an acute inferior ST elevation myocardial infarction (STEMI).

## Case presentation

A 47-year-old obese Caucasian woman with a history of thyroid cancer, hypothyroidism, depression, and tobacco abuse presented to the hospital with acute chest discomfort. She described a substernal, diffuse, nonradiating heavy sensation over her chest that was a 7 out of 10 in severity. She was diaphoretic and nauseous, and reported no prior similar episodes.

Her social history was significant for a 20-pack-year smoking history and occasional alcohol use. She works as a real estate agent, does not exercise, and is relatively sedentary most of the day. Our patient denied any history of toxic exposure or recent travel. Her family history was significant for cardiovascular disease in her grandfather, who died in his 70s from a heart attack. Home medications included oral levothyroxine (137 mcg once daily) for hypothyroidism and oral bupropion (150 mg once daily) for depression. She denied any symptoms of claudication and her review of systems was noncontributory.

Her vital signs revealed a blood pressure of 102/59 mm Hg, a heart rate of 49 beats/minute, temperature of 98.6 °F, and oxygen saturation of 97% on room air. Her body mass index (BMI) was 37 kg/m^2^. Our patient appeared to be in moderate distress. A neck examination showed no lymphadenopathy, jugular venous distension, or carotid bruits. On cardiac examination, she had a slow heart rate, regular rhythm, normal S1 and S2, and no murmurs, rubs, or gallops were appreciated. Her breath sounds were clear and symmetric bilaterally without any crackles, wheezes, or rhonchi. Her abdomen was soft, nondistended, and nontender with normal bowel sounds and no organomegaly. Extremities showed no lesions, rashes, or lower extremity edema. Radial pulses were 2+ bilaterally, femoral pulses 2+ bilaterally, posterior tibial pulses 1+ bilaterally, and dorsalis pedis pulses absent bilaterally. On neurological examination, our patient was awake, alert, and oriented to person, place, and time. Her cranial nerves 2–12 were grossly intact, motor strength was 5 out of 5 throughout, sensation was intact to soft touch and pinprick throughout, and her gait was not assessed.

An electrocardiogram showed sinus bradycardia with ST-elevation in inferior leads II, III, and aVF, as well as reciprocal ST depression in leads I and aVL – suggestive of an acute inferior STEMI. Our patient was given intravenous heparin (5000 mg), oral aspirin (325 mg), and oral ticagrelor (180 mg), and immediately taken to the catheterization laboratory.

Laboratory workup revealed a white blood cell count of 12,300/mm^3^, hemoglobin of 14.4 g/dL, and platelet count of 262,000/mm^3^. A chemistry panel showed a sodium of 139 mmol/L, potassium of 4.4 mmol/L, carbon dioxide of 15 mmol/L, blood urea nitrogen (BUN) of 13 mg/dL, creatinine of 0.8 mg/dL, international normalized ratio (INR) of 1.18, and troponin of < 0.012 ng/mL. Liver function tests were within normal limits.

**Fig. 1:**
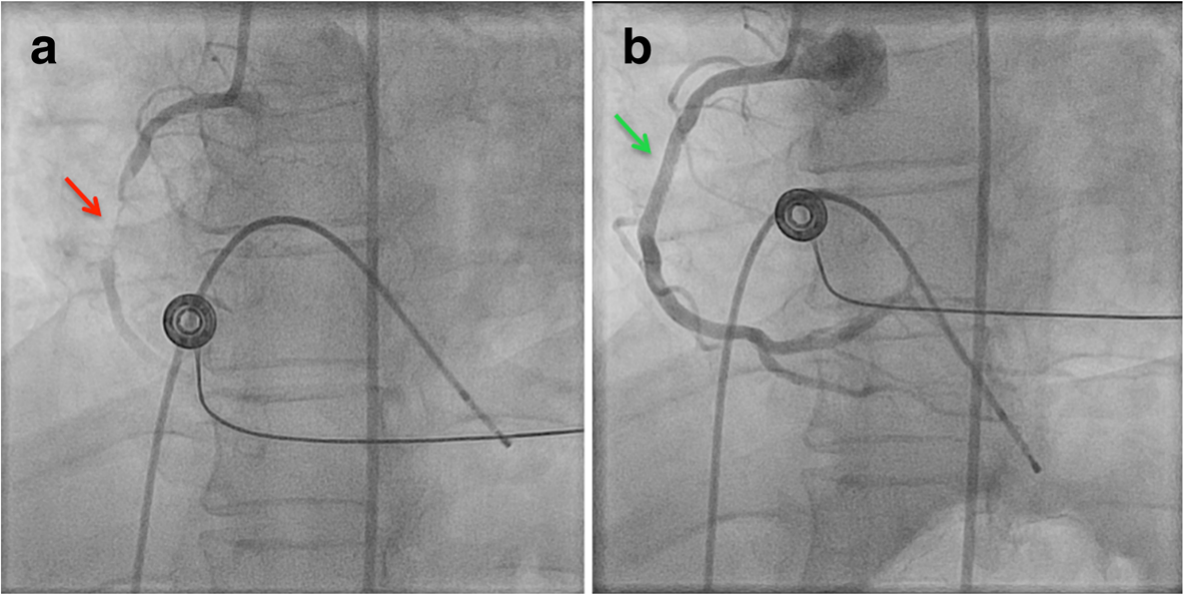
Coronary angiography showing a subtotally occluded right coronary artery in its mid-portion (*red arrow*) (**a**). Right coronary artery after a 3.0 × 28 mm Promus Premier stent was deployed, showing a significant improvement in blood flow (*green arrow*) (**b**)

Left heart catheterization and coronary angiography were performed via the right femoral artery. There was difficulty obtaining the vascular sheaths by the right femoral artery, which was likely from preexisting peripheral vascular disease. Left ventriculography showed inferior hypokinesis with an ejection fraction of approximately 55%. Coronary arteriography revealed a 60% proximal diagonal branch lesion. The right coronary artery was subtotally occluded in its mid-portion (Fig. 1a). A 3.0 × 28 mm Promus Premier stent was deployed with significant improvement in blood flow (Fig. 1b). Our patient suddenly became uncomfortable and reported severe bilateral lower extremity pain. Her femoral pulses were diminished and could not be palpated in either leg – even with Doppler. Both lower extremities were pale and cool to touch. An aortography showed no dye flow below L1–L2. The vascular surgeon was called and our patient was immediately taken to the operating room.

**Fig. 2:**
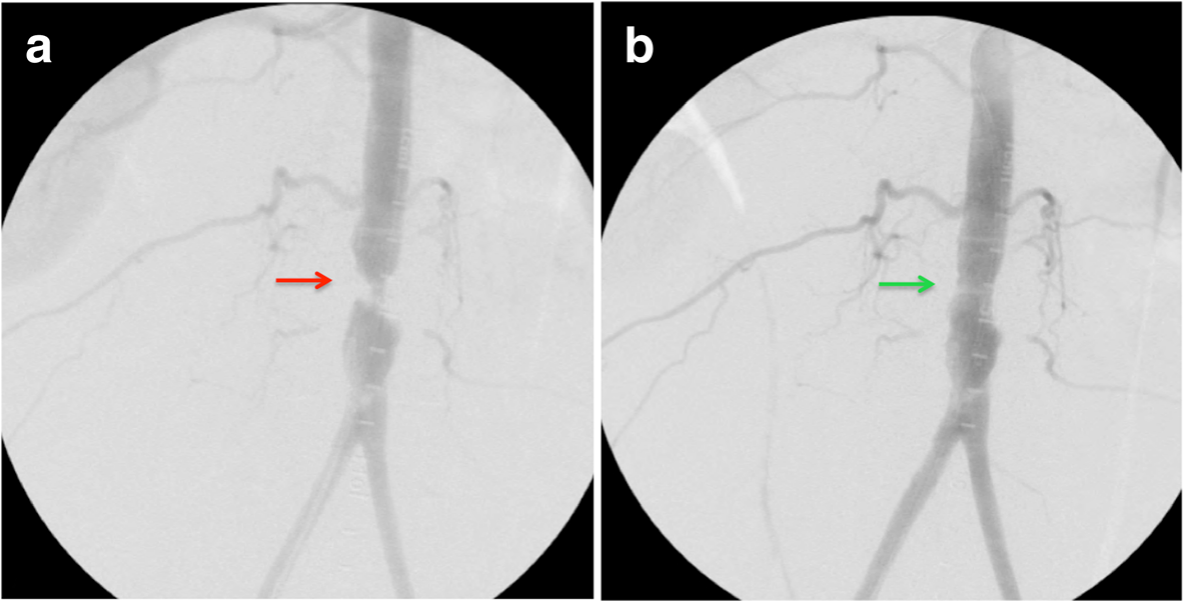
Distal aortogram showing 95% stenosis with an apple core-like lesion in the mid-abdominal aorta (*red arrow*) (**a**). Distal aortic segment after an Epic self-expanding stent was deployed, demonstrating a significant improvement of blood flow through the distal aortic segment (*green arrow*) (**b**)

**Fig. 3:**
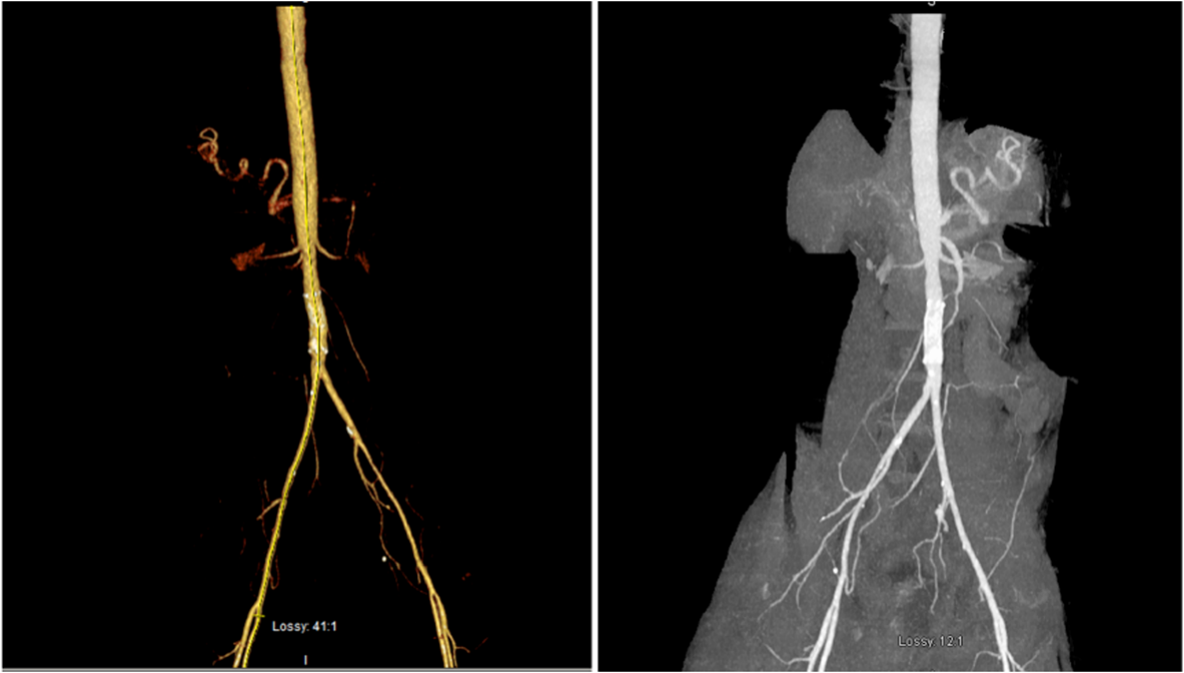
Computed tomography angiography with runoff showing the stented segment of the abdominal aorta widely patent without evidence of significant stenosis or occlusion

A distal aortogram revealed 95% stenosis with an apple core-like lesion in the mid-abdominal aorta (Fig. 2a). An Epic self-expanding stent was deployed, resulting in significant improvement of blood flow through the distal aortic segment (Fig. 2b). The iliac artery was small but patent. After stent placement, our patient’s pain resolved and distal pulses became palpable. The rest of the hospital course was unremarkable and our patient was discharged 48 hours after the procedure. Our patient remained asymptomatic on follow-up 6 months later. Computed tomography (CT) angiography with aortic runoff showed the stented segment of the abdominal aorta remained widely patent without evidence of significant stenosis or occlusion (Fig. 3).

## Discussion and conclusions

This report demonstrates an unusual case of simultaneous type I acute MI and aortoiliac occlusive disease. It is likely that this patient had preexisting severe peripheral vascular disease that was never diagnosed or symptomatic because of her relatively sedentary lifestyle. Her history of tobacco abuse and obesity most likely contributed as well. It was only a matter of time until these factors led to her symptomatic presentation. Unfortunately, it was only during the percutaneous transluminal angioplasty that the severity of her vascular disease was discovered.

We believe that there are two leading hypotheses for the development of her acute aortoiliac occlusion in the setting of STEMI. Both reasonably assume that our patient had preexisting severe peripheral vascular disease, as evidenced by the difficulty in gaining femoral access during angioplasty. The first possibility is that the stressful insult of the STEMI precipitated rupture of a preexisting plaque, leading to sudden aortoiliac occlusion. The second possibility is that it was iatrogenic. In other words, the catheters used during the procedure may have dislodged a thrombus triggering immediate aortoiliac occlusion; or, the catheters traveling through an already severely stenosed abdominal aorta may have caused complete obstruction and the immediate symptoms distally.

Ideally, a routine angiogram with runoff views before cardiac catheterization and intervention would be the best option in patients with high suspicion of peripheral vascular disease. However, this approach may not always be practical, especially in the setting of an acute STEMI when time is a critical factor.

In closing, there are two important thoughts we leave for interventional cardiologists to consider. The first is to be cautious when manipulating the wire and catheter at all times and, even more so, in patients with history of peripheral vascular disease or risk factors that suggest its presence. In this case, the only significant risk factors were the patient’s obesity, tobacco abuse, sedentary lifestyle, and acute STEMI presentation. While there was no reported history of claudication and femoral pulses were intact on presentation, distal lower extremity pulses were diminished. The diminished distal lower extremity pulses should be a clue for the presence of preexisting peripheral vascular disease and caution wire and catheter manipulation. Even though it is still unclear what precipitated this patient’s aortoiliac occlusion, it is likely that the actual catheter caused a physical obstruction of an already severely stenosed aorta.

Second, in patients with known or suspected peripheral vascular disease or when there is difficulty with femoral access during percutaneous transluminal angiography, interventional cardiologists should consider a transradial approach. This allows for bypassing the abdominal and thoracic aorta, thereby lowering the risk of distal occlusion and embolization. While this latter suggestion has not been studied, a recent literature review suggests a reduction in complications and hospital mortality with the transradial approach when compared to the transfemoral approach [6]. Therefore, in patients with evidence of peripheral vascular disease, interventional cardiologists should (1) take extra caution when manipulating the wire and catheter and (2) consider using the radial artery as an alternative approach.

## Abbreviation

STEMI: ST-elevation myocardial infarction
